# Identification of *Plasmodium falciparum* nuclear proteins by mass spectrometry and proposed protein annotation

**DOI:** 10.1371/journal.pone.0205596

**Published:** 2018-10-31

**Authors:** Sylvie Briquet, Asma Ourimi, Cédric Pionneau, Juliana Bernardes, Alessandra Carbone, Solenne Chardonnet, Catherine Vaquero

**Affiliations:** 1 Sorbonne Université, INSERM, CNRS, Centre d'immunologie et des maladies infectieuses, CIMI-Paris, Paris, France; 2 Sorbonne Université, INSERM, UMS Omique, Plateforme Post-génomique de la Pitié-Salpêtrière, Paris, France; 3 Sorbonne Université, CNRS, IBPS, Laboratoire de Biologie Computationnelle et Quantitative (LCQB), 4 place Jussieu, Paris, France; 4 Institut Universitaire de France, Paris, France; Institut national de la santé et de la recherche médicale - Institut Cochin, FRANCE

## Abstract

The nuclear proteome of *Plasmodium falciparum* results from the continual shuttle of proteins between the cell cytoplasm—nucleus and *vice versa*. Using shotgun proteomics tools, we explored the nuclear proteins of mixed populations of *Plasmodium falciparum* extracted from infected erythrocytes. We combined GeLC-MS/MS and 2D-LC-MS/MS with a peptide ion exclusion procedure in order to increase the detection of low abundant proteins such as those involved in gene expression. We have identified 446 nuclear proteins covering all expected nuclear protein families involved in gene regulation. All structural ribosomal (40S and 60S) proteins were identified which is consistent with the nuclear localization of ribosomal biogenesis. Proteins involved in the translation machinery were also found suggesting that translational events might occur in the nucleus in *P*. *falciparum* as previously hypothesized in eukaryotes. These data were compared to the protein list established by PlasmoDB and submitted to Plasmobase a recently reported *Plasmodium* annotation website to propose new functional putative annotation of several unknown proteins found in the nuclear extracts.

## Introduction

In eukaryote cells, the nucleus is a highly dynamic and complex organelle [1] [2] where major regulatory gene expression events take place such as DNA replication, RNA synthesis within transcriptional machinery, mRNA processing and transport to the cytoplasm as well as ribosomal sub-units biogenesis. The nucleus is also organized to participate in RNA, protein and ribosomal sub-unit trafficking in and out of the nucleus [3]. In *Plasmodium*, the dynamic organization and function of the nucleus vary throughout the different stages of cell development from active multiplication to parasite differentiation. Diverse sub-proteomes of the nucleus such as nucleolus or nuclear membranes have been investigated in eukaryotes by a number of proteomics experimental procedures including 2D gels, 1D gels followed by LC-MS/MS (GeLC-MS/MS), direct 2D-LC-MS/MS shotgun analysis or a combination of these approaches. A combination of both experimental approaches enhances the coverage compared to either individual methods [4]. However, a comprehensive identification of nuclear proteins still awaits completion in eukaryotes. Of note, a continuous cytoplasm-nucleus protein shuttling occurs in eukaryotes weakening the characterisation and definition of nuclear proteins even though proteins accumulate either in the cytoplasm or in the nucleus according to the cellular development [5]. This back and forth protein transport through the nuclear pore plays an important role in the control of gene expression.

*Plasmodium falciparum* is a parasite responsible for the most pathogenic malaria with around 500 000 malaria deaths (range 236000–635000) per year mostly in African countries, mostly comprising children under five years and pregnant women (WHO 2015). The genome of the parasite is extremely AT-rich from 80% in coding regions to 90% in intergenic and promoter regions. Among the ~ 5500 predicted open reading frames, about 50% are not assigned to putative functions. For *Plasmodium* parasites, DNA genomic sequences, open reading frame prediction and protein annotation are under constant curation in PlasmoDB. Even though the *Plasmodium* community participates actively to the comprehension of the parasite complex cell cycle, only a small number of proteins was functionally investigated most of them implicated during invasion of erythrocytes and hepatocytes by merozoites and sporozoites, respectively. Previous proteomics analyses were performed in whole parasite extracts prepared from various life stages all throughout the erythrocytic development (rings to schizonts; gametocytes and sporozoites) [6] [7] [8] [9] or from parasite sub-fractions [10] [11]. The parasite proteome was also investigated under drug treatment [12]. Only one study focussed on the nuclear proteome using shotgun LC-MS/MS [13] at different stages of erythrocytic parasite development (ring, trophozoite and schizont).

Here, we explored the nuclear protein content of mixed populations of 3D7 *P*. *falciparum* from parasitized red blood cells (pRBC). We decided not to focus on the dynamic changes in the nuclear protein composition during the erythrocytic cycle. Our main objective was the identification of nuclear proteins associated to gene regulation including proteins involved in DNA replication, mRNA synthesis, maturation and transport to the cytoplasm as well as proteins involved in translation such as ribosomal proteins [14] and translational factors [15]. The difficulty of protein determination resides mostly in the low abundance of numerous eukaryote nuclear proteins. To this end, we used a combination of 2D-LC-MS/MS with precursor ion exclusion (PIE) procedure and GeLC-MS/MS. These two complementary approaches allowed the identification of 446 proteins with a high rate of proteins ascribed to nuclear compartment and functions. Moreover, we took advantage of the just new released website Plasmobase [16] to assign new domain architectures that had not been reported yet in *Plasmodium* [17]. Our results bring an overall improvement in *Plasmodium* nuclear protein annotations.

## Material and methods

### *Plasmodium falciparum* culture

The 3D7 clone of *P*. *falciparum* was provided by D. Walliker. Venous blood from informed healthy donors was obtained from the French blood bank institute (EFS) according to the agreement between INSERM and EFS (CPSL C UNT—15/EFS/012). During the medical examination preceding blood donation, the medical doctor of EFS informed the healthy donors that part of their blood could be used for research. All blood samples used in the present study have been provided by healthy donors, which signed the agreement. All blood samples were deidentified and anonymized at the EFS prior deliverance to the laboratory. According to the French law (L1211-2), this research is considered as a non-interventional research that does not require prior approval of the ethics committee. The red blood cells were grown asynchronously as described by [18] except that the culture medium RPMI 1640 (Gibco Invitrogen) was supplemented with 0.5% Albumax I (Gibco Invitrogen). Parasitaemia was daily monitored on Giemsa-stained blood smears with regular medium replacement.

### Preparation of the biological materials: Nuclear and cellular extracts

Nuclear extracts (NE) and cytoplasmic extracts (CE) were prepared from 50 ml of red blood cells at 5% parasitaemia infected with 3D7 asexual-stage cells, as described by Osta *et al*. [19].

Cells were lysed in PBS containing 0.13% saponin. The parasites were pelleted at 1200 g, 5 min, then washed with Buffer I (0.34 M sucrose, 15 mM NaCl, 0.5 mM spermidine, 0.15 mM spermine, 0.2 mM EDTA, 0.2 mM EGTA 15 mM, Tris-HCl pH 7.4 and 0.2 mM PMSF). In order to eliminate the maximum of all human globins from the NE preparation, we added extra washes to the parasite purification with Buffer I prior to nuclear extraction.

The parasite pellet was resuspended in Buffer I containing 1% Triton X-100 and homogenized with 20 strokes in a Dounce homogenizer (B pestel). The nuclei were pelleted at 600 g for 5 min and upper part of the supernatants corresponding to CE were sampled, frozen in liquid nitrogen and conserved at -80°C. Then the nuclear pellet was washed once with Buffer I and resuspended in a volume of low salt buffer [1.5 mM MgCl^2^, 0.2 mM EDTA, 20 mM HEPES pH 7.9, 25% glycerol and protease inhibitor cocktail (Roche)] equal to half the packed nuclear volume. A volume of high salt buffer (low salt buffer containing 1.2 M KCl) equal to half the packed nuclear volume was then added dropwise and the nuclei were extracted for 30 min with gentle mixing at 4°C. The extracted nuclei were centrifuged at 12000 g for 30 min at 4°C. The supernatants containing the NE were stored at -80°C.

Protein quantification was obtained by Bradford assay (Bio-Rad). To examine the quality of the various nuclear extract preparations, 10 μg of NE1 and NE2 as well as their corresponding cellular extracts (CE1 and CE2), were separated by SDS-PAGE on a 12% gels stained with Coomassie blue.

### Western blot analysis

Ten μg of NE or CE and 100 ng of recombinant protein PfHMGB2 used as positive control were run on 12% gel electrophoresis SDS-PAGE and subjected to Western blotting experiments after transfer onto polyvinylidene difluoride (PVDF) membranes (Bio-Rad). The PVDF membrane was cut in two pieces at the level of 25 kDa. After blocking with ODYSSEY buffer (Biosciences) diluted ½ in PBS for 12 hr, the upper part of the membrane was probed with the primary antibody (AB): goat anti-aldolase, #AB1809 Chemicon International (1/5000) and the lower part by the rabbit anti-*Pf*HMGB2, a gift from V. Chauhan from ICGEB, New Delhi (1/20000) diluted ½ in PBS + 0.1% Tween. After 3 hr incubation the membranes were washed three times 15 min with PBS + 0.1% Tween, followed by incubation with donkey anti-goat IgG alexa fluor 800 conjugate #W10825 Thermo Fischer (1/10000) and goat anti–rabbit IgG alexa fluor 680 conjugate #A27042 Thermo Fischer (1/10000) and revealed by ODYSSEY scanner device (Li-Cor Biosciences), respectively. Orange colour stands for aldolase revelation and green colour for the *Pf*HMGB2.

### GeLC-MS/MS

#### SDS-PAGE

Protein mixtures samples (15–20 μg) were solubilised in SDS-PAGE Laemmli buffer and separated by SDS-PAGE on a 12% gel. Gels were fixed with 50% ethanol, 7% acetic acid for 2hr and stained with SyproRuby (Invitrogen) and 24 or 32 gel bands were manually excised, depending on the complexity of the samples.

#### In-gel digestion

Gel sections were washed with [25 mM ammonium bicarbonate (AmBic), 50% acetonitrile (ACN)] then dehydrated in ACN and submitted to reduction [10 mM dithiothreitol (DTT) in 100 mM AmBic for 30 min at 56°C] and alkylation [55 mM iodoacetamide (IAM) in 100 mM AmBic for 30 min, RT]. After dehydration with ACN, digestion was performed with 200 ng trypsin per band overnight at 37°C in 50 mM AmBic, 10% ACN. Supernatants containing peptides were collected in new tubes. Band pieces were washed twice in 60% acetonitrile, 0.1% TFA for 10 min in an ultrasonic bath and supernatants were pooled for each sample. Peptides were completely dried in SpeedVac (Thermo Savant), then solubilized in 4 μl of 0.1% trifluoroacetic acid (TFA), 30% ACN and diluted with 26 μl of 0.1% formic acid to reduce the acetonitrile concentration to 4%.

#### LC-MS/MS analysis

Peptides separations were performed on an Ultimate3000 HPLC (Thermo). Tryptic peptides were loaded onto a trap column (PepMap100 C18, 5 μm, 100 Å, 300 μm ID, 5 mm) using buffer A (flow: 20 μl/min) and separated using reverse-phase C18 analytical column (PepMap100 C18, 3 μm, 100 Å, 75 μm i.d., 15 cm) with a linear gradient of buffer B (95% acetonitrile, 4.9% H2O, 0.1% formic acid) ranging from 0 to 50% within 70 min (flow: 300 nl/min). Eluted peptides were analysed by nanoESI ion trap mass spectrometer (HCTultra, Bruker) set to isolate and fragment the top 5 most abundant precursors. Electrospray voltage was set to 2000V and analysis mass ranges were 250–1500 m/z for MS and 200–3000 m/z for MS/MS. DataAnalysis software version 3.4 from Bruker was used to generate mgf files from raw data, with a precursor intensity threshold of 3.10^5^. The mgf files were submitted to local Mascot server using both PlasmoDB v13 (14 jan 2015, 5542 entries) and human SwissProt (6 march 2013, 20329 entries) databases. Search parameters were the following: MS and MS/MS mass tolerance = 0.5 Da; missed cleavages = 1; fixed modification = Carbamidomethyl (C), variable modification = Oxidation (M): FDR < 1% (search against the reversed merged database).

### 2D-LC-MS/MS shotgun analyses

#### In-solution digestion

Ten μg of all nuclear and cytoplasmic extracts were first reduced by adding 10 mM DTT in 100 mM AmBic for 30 min at 56°C, and then alkylated with 55 mM IAM in 100 mM AmBic. Samples were subjected to trypsin digestion (protein/trypsin ratio: 50/1) for 16 hr at 37°C. Peptides mixtures were completely dried on the SpeedVac (Thermo Savant) and reconstituted in 10 μl of buffer A (99.9% H2O, 0.1% formic acid).

#### 2D-LC-MS/MS

Peptides separations were performed on an Ultimate3000 HPLC (Thermo). One μg of peptide mixture was loaded onto a strong cation exchange (SCX) column (BioX-SCX, 5 μm, 500 μm i.d., 15 mm) using buffer A (flow: 20 μl/min) and eluted with 11 consecutive ammonium acetate solutions with stepwise increasing concentration (5mM, 10 mM, 15m M, 20 mM, 35 mM, 50 mM, 75m M, 100 mM, 250 mM, 500 mM and 1000 mM). Each SCX fraction was on-line transferred to a Reverse Phase (RP) column (PepMap100 C18, 3 μm, 100 Å, 75 μm i.d., 15 cm) and peptides were eluted with a linear gradient of buffer B (95% acetonitrile, 4.9% H2O, 0.1% formic acid) ranging from 0 to 50% within 60 min (flow: 300 nl/min). Eluted peptides were analysed by nano ESI ion trap mass spectrometer (HCT ultra, Bruker) set to isolate and fragment the top 6 most abundant peptides per cycle with a 30 sec dynamic exclusion time. Electrospray voltage was set to 2000 V and analysis mass ranges were 250–1500 m/z for MS and 100–3000 m/z for MS/MS. Data analysis was performed as described for GeLC-MS/MS. Considering the remaining high amounts of human globins in the samples, leading to wide chromatographic peaks, the dynamic exclusion time was extended to 150 sec. The corresponding protein list obtained with the previous parameters but with the extended dynamic exclusion time of 150 sec was named Without Exclusion (WE). Moreover, we improved the dynamic exclusion process by adding precursor ion exclusion (PIE) mass lists of the most abundant proteins including human globins and albumin in order to favour the identification of low abundance proteins [20] [21]. As the MS controlling software (Esquire Control v6.1) allowed the exclusion of 39 masses at once, we first defined exclusion mass lists for each salt fraction (5 mM, 10 mM, 15 mM, 20 mM, 35 mM, 50 mM, 75 mM, 100 mM, 250 mM, 500 mM, et 1 M) accounting for a total of 39x11 = 429 excluded precursors per sample (fraction PIE: F.PIE). In a second time, we divided LC-MS/MS runs into five retention time windows [15 min-30 min], [30 min-40 min], [40 min-50 min], [50 min-60 min], [60 min-80 min] to enable the exclusion of 39 precursors per retention time windows, meaning 39x5 = 195 excluded precursors per salt fraction, leading to a possibility of 39x5x11 i.e. 2145 excluded precursors per sample (enhanced PIE: E.PIE), see the schematic procedures in S1 Fig. The mass spectrometry proteomics data have been deposited to the ProteomeXchange Consortium via the PRIDE partner repository with the dataset identifier PXD010427.

### Gene ontology analysis

A gene ontology analysis of nuclear extracts was performed using PANTHER (version 13.1 Released 2018-02-03) [22]. To this end, PlasmoDB gene names were converted into UniProt accession numbers using Retrieve/ID mapping tool in UniProt. Both CE and NE were analysed using the functional classification pie chart” for Protein Classes (molecular function) and Organelle (subcellular localisation) GO terms using *P*. *falciparum* organisms. PANTHER overrepresentation test (released 2017 1205) was also used to highlight the functional biological processes statistically overrepresented in NE compared to the complete *P*. *falciparum* gene data base (Fischer test, p value < 0.01, FDR < 5%).

### Annotation of the unknown proteins

We used Plasmobase [16], a new online resource proposing domain architectures of proteins for 11 *Plasmodium* species. In Plasmobase (http://genome.lcqb.upmc.fr/plasmobase/), domain architecture reconstruction relies on a new method of architecture prediction, DAMA and on a new annotation strategy of domain prediction, CLADE [17], based on a multi-source modelling approach. Plasmobase significantly increases the Pfam domain coverage by proposing new domain architectures and new functional protein annotation. Moreover, Plasmobase allows the user to compare protein domain architectures found in the 11 *Plasmodium* fully sequenced genomes present in PlasmoDB, the reference repository for *Plasmodium* species. Plasmobase proposes a visualisation of domain architectures in all *Plasmodium* complete genomes and allows an easy comparison among architectures in *Plasmodium* species *versus* all species of Uniprot. In particular, it is possible to determine if a specific domain architecture is conserved within the *Plasmodium* species, and to explore it in both Plasmobase and UniProt Database, a curated collection of manually evaluated and functionally annotated eukaryotic proteins, for proposing the same annotation to the corresponding *Plasmodium* proteins.

## Results

### Identification of *P*. *falciparum* nuclear proteins using GeLC-MS/MS

**Fig 1:**
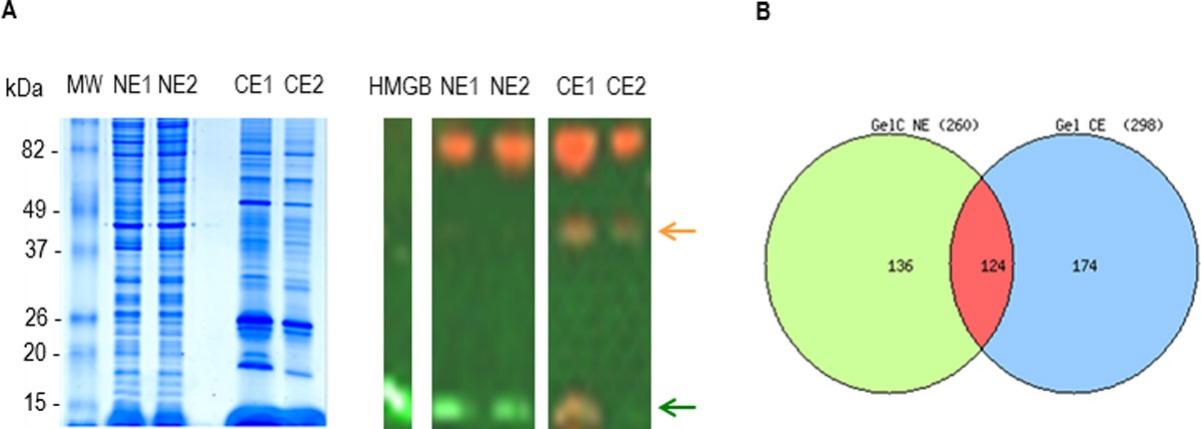
Comparison between two nuclear and cytoplasmic extracts. **A.** SDS-PAGE of 10 μg of two nuclear biological replicates NE1 and NE2 as well as their corresponding cytoplasmic extracts CE1 and CE2 were stained by Coomassie Blue (left). For the Western blotting experiment (right) the membrane was probed by an anti-aldolase and an anti-*Pf*HMGB2 revealing the corresponding proteins; orange arrow for aldolase and green arrow for *Pf*HMGB2. HMGB corresponds to 100 ng of recombinant *Pf*HMGB2 used as a control. **B.** Venn diagram of the proteins identified in CE and NE extracts (the list of proteins is reported in S2 Fig).

Our aim was to identify *P*. *falciparum* nuclear proteins with a special focus on all proteins involved in overall gene regulation from DNA replication to RNA transcription [23], mRNA maturation and transport as well as mRNA translation. It is well documented that the level of transcript and protein expression varies all throughout the erythrocytic parasite development [24]. For comprehensive identification of *P*. *falciparum* nuclear proteins we decided to prepare nuclear extracts from mixed populations of parasites encompassing ring to schizont stages. To validate the quality of our nuclear extracts, the content of nuclear (NE1 and NE2) and corresponding cytosolic extracts (CE1 and CE2) was evaluated by SDS-PAGE. Migration profiles showed clear differences (Fig 1A, left part). Using WB, we show that the cytosolic protein aldolase was detected in CE only, suggesting very low contamination of the nuclear extracts by cytosolic proteins. An unspecific protein was also revealed by the anti-aldolase. In parallel, *Pf*HMGB2, an abundant nuclear chromatin remodelling factor, was found abundant in NE suggesting a good enrichment in nuclear proteins. Notably, *Pf*HMGB2 has been shown to be also present in the cytoplasm [24], explaining the band detected in CE. Thus, the WB profiles exactly correspond to the expected subcellular location of both proteins showing the good quality of the nuclear extracts.

We then compared the protein content of a given nuclear extract to its corresponding cytoplasmic extract by a classical GeLC-MS/MS approach. The resulting Venn diagram of both extracts is presented in Fig 1B. Within the 260 proteins identified in the nuclear extract, 136 were found only in the nucleus and 124 were shared with the cytoplasm. The nuclear extract was clearly enriched [Pivot Table (PT) in S2 Fig] in proteins assigned to nuclear functions including all abundant epigenetic proteins (histones, nucleosome assembly and remodelling proteins like HMGB proteins). Splicing factors and several proteins involved in DNA replication and RNA transcription were also specifically detected in nuclear extract. On the other hand, cytoplasmic proteins were clearly under-represented, such as Ras/Rab related proteins, many proteasome sub-units and cellular enzymes as well as heat shock proteins. For instance, Hsp60 recognised as a genuine cytoplasmic protein was not detected in nuclear extracts. These data highlight the quality of our subcellular fractionation. Regarding the shared proteins, ribosomal [14] and translation proteins [25], as well as several proteasome sub-units, heat shock proteins and proteins involved in DNA and RNA gene regulation were found in both extracts. The presence of most of these proteins in both compartments was expected and this point will be addressed in the Discussion.

### Identification of *P*. *falciparum* nuclear proteins using 2D-LC-MS/MS with PIE

**Fig 2:**
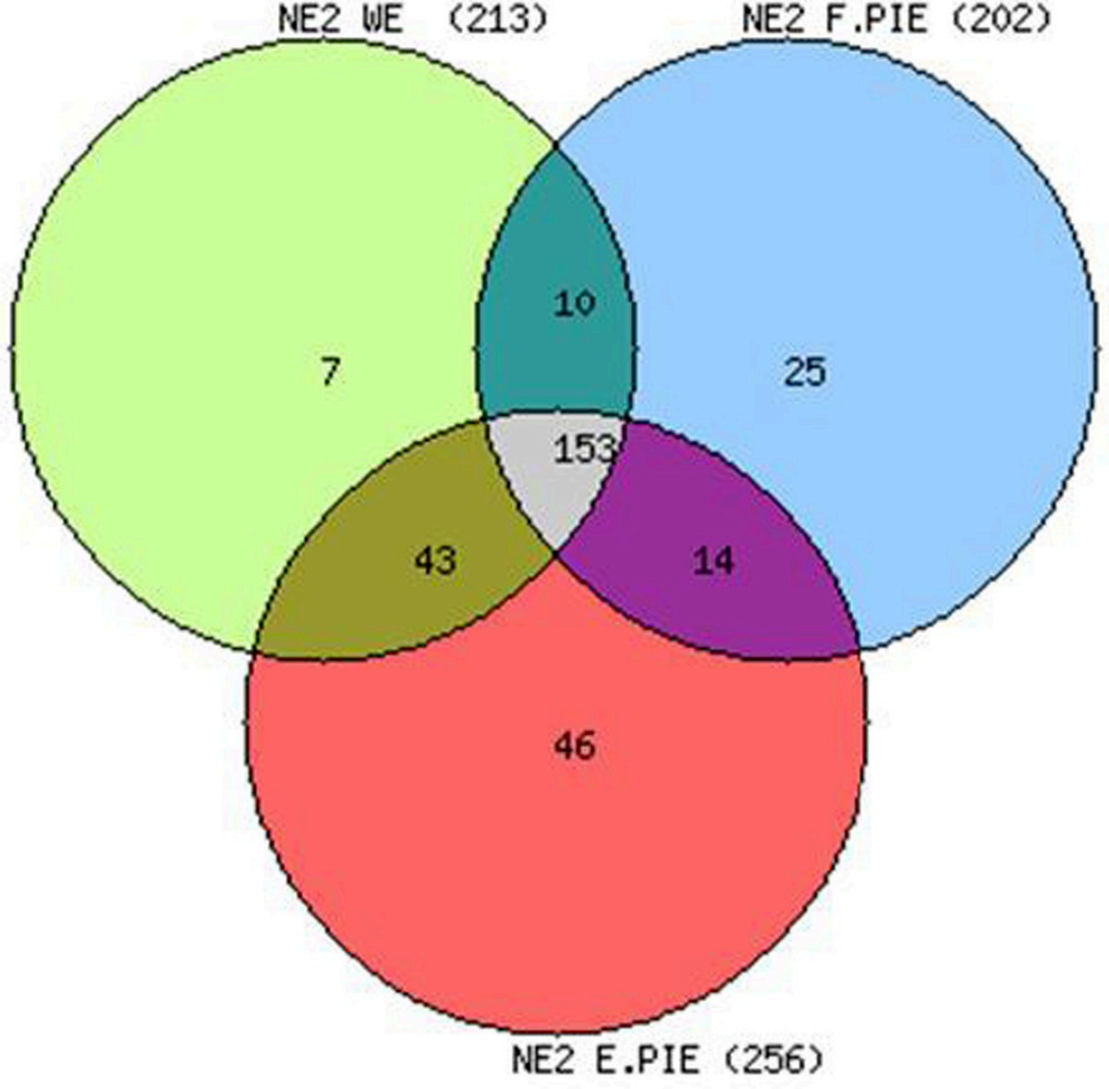
Progression of the number of identified proteins by precursor ion exclusion. Venn diagram of three technical repeats of one μg of NE2 analysed by 2D-LC-MS/MS either without exclusion (WE) or after precursor ion exclusion F.PIE and C.PIE as described in Methods and S1 Fig.

We performed a complementary proteomic approach based on strong cation exchange (SCX) fractionation applied on tryptic peptides with the aim of expanding our list of nuclear proteins. Peptides were separated by SCX followed by RP chromatography using 11 salt steps for SCX fractionation, ranging from 0 to 1000 mM ammonium acetate as described in M&M. Preliminary experiments were run to optimize data acquisition parameters. Injection of 1 μg of protein extract (dose response experiments from 0.5 to 2 μg) with a dynamic exclusion time of 150 sec gave the highest rate of identification (data not shown). Because of stochastic precursor selection inherent to data-dependant acquisition LC-MS/MS, three successive runs (technical replicates) were performed for each sample. Using these parameters corresponding to the Without Exclusion procedure (WE) we obtained a list of 213 *Plasmodium* proteins from nuclear extract NE2 (Fig 2).

Of note, despite the use of extensive purification for nuclear extract preparations, human globin proteins remained highly abundant in our samples and prevented identification of low abundance proteins. To overcome this limitation, we implemented Precursor Ion Exclusion (PIE). The ion exclusion lists were designed to prevent the selection and the fragmentation of the most abundant peptides identified in the first 2D LC-MS/MS WE run. In a first step, 39 precursor ions were excluded per salt fraction (F.PIE for Fraction PIE) leading to a maximum of 429 ion exclusion. This strategy allowed the identification of 39 additional proteins not detected in the WE experiment (Fig 2). To further deepen our analysis, we split each LC-MS/MS acquisition salt fraction run into 5 consecutive retention time windows which enabled the theoretical exclusion of up to 195 precursors per salt fraction. Therefore, 1071 abundant ions were excluded out of the 2145 theoretical exclusion possibilities (E.PIE for Enhanced PIE). Using E.PIE, we gained another 46 proteins. The S3 Fig highlights the increase of proteins in F.PIE and E.PIE never detected in WE. Among them, several proteins known to be involved in gene regulation: histone H3, chromodomain and DEAD/DEAH helicases, an ApiAP2 as well as several additional 40S and 60S ribosomal and translational proteins were identified. Altogether, the combination of 2D LC-MS/MS WE, F.PIE and E.PIE allowed the identification of 298 *P*. *falciparum* proteins for one nuclear extract. Each acquisition mode was repeated using several independent nuclear preparations leading to the identification of a total of 368 *P*. *falciparum* proteins.

### Analysis of all identified proteins obtained with the two approaches

**Fig 3:**
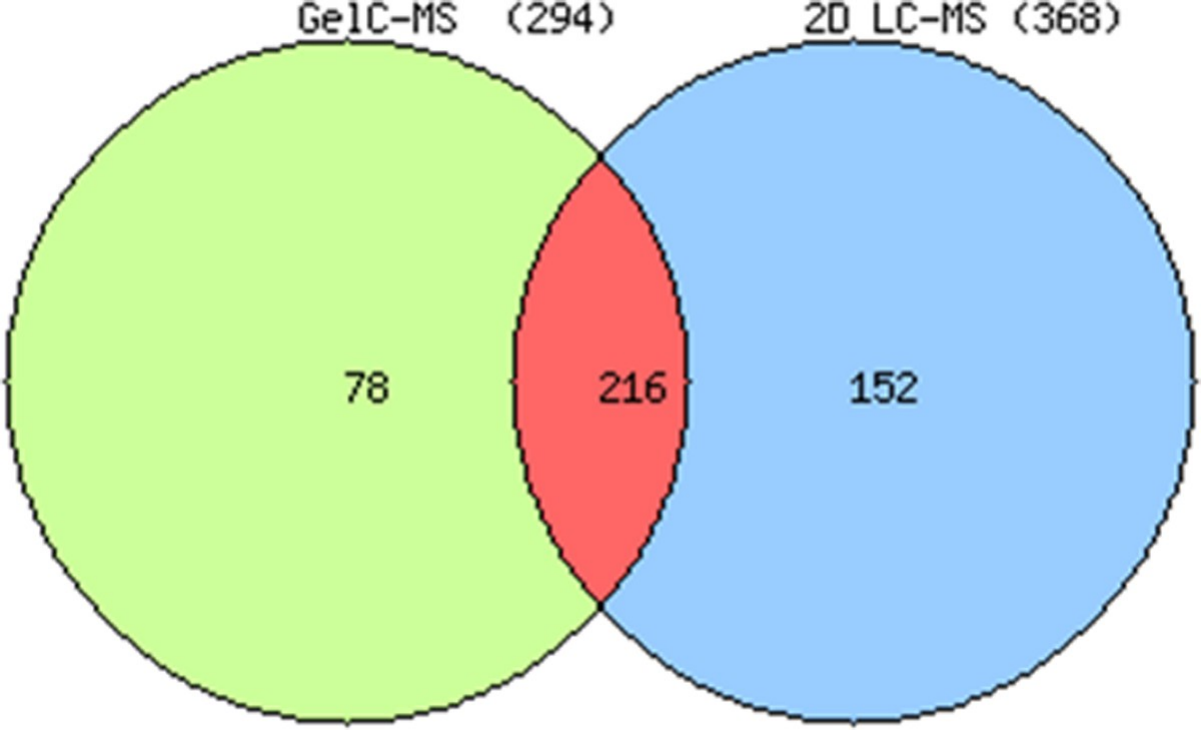
Venn diagram of all proteins identified by GeLC-MS/MS and 2D-LC-MS/MS. Venn diagram of several biological and technical nuclear extract replicates analysed either by GeLC-MS/MS or 2D-LC-MS/MS in order to generate a complete list of 446 identified proteins presented in S4B Fig.

A total of 368 proteins were identified by 2D-LC-MS/MS performed on several nuclear extract replicates that were compared to the proteins identified (294) with the GeLC-MS/MS. Fig 3 shows that among the 446 total unique *Plasmodium* proteins, 216 were shared by the two experimental strategies whereas 78 and 152 were only detected in GeLC-MS/MS and 2D-LC-MS/MS, highlighting the complementarity of these two approaches (see S4A Fig.). Even though several 2D-LC-MS/MS technical and biological replicate analyses were achieved some proteins were only detected by GeLC-MS/MS including DNA replication factors, members of DNA polymerase II, RNA helicases and binding factors as well as several translational proteins.

**Fig 4:**
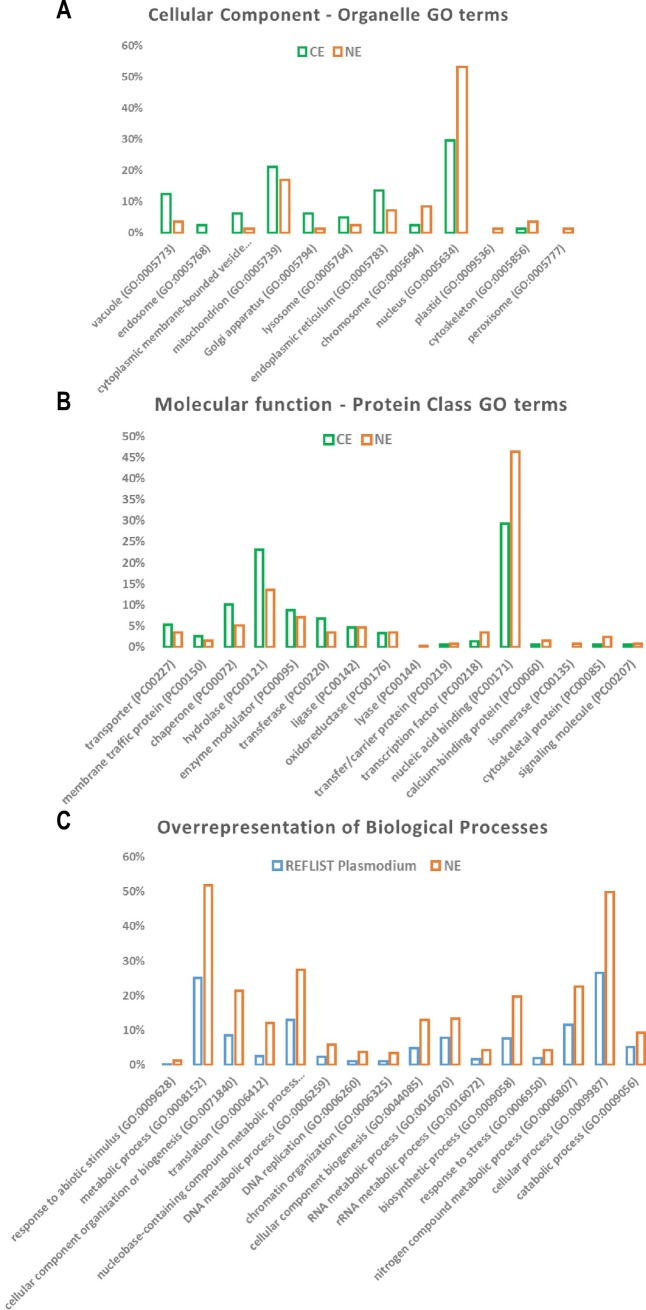
Gene ontology analysis. GO term analysis was performed using PANTHER functional classification. **A.** Organelle GO terms distribution for CE and NE (percent of the total number of hits). **B.** Protein Class GO terms illustrating the relative proportion of protein molecular functions in CE and NE (percent of the total number of hits). **C.** Biological processes significantly overrepresented in NE compared to the complete *P*. *falciparum* gene database (Fischer test, p value < 0.01, FDR < 5%).

From the complete list of 446 proteins (see Pivot Table S4B Fig), we performed a Gene Ontology analysis to highlight the protein categories overrepresented in NE. PANTHER [22] was used to assess subcellular organelle distribution and molecular function (protein class) in CE and NE (S5 Fig). Nuclear and chromosome associated proteins are clearly more abundant in NE compared to CE (Fig 4A). Similarly, nucleic acid binding proteins and transcription factors are enriched in NE (Fig 4B). In addition, biological processes overrepresentation was measured using PANTHER algorithm, showing significant enrichment in DNA metabolic process, DNA replication, chromatin organization and RNA metabolic process (Fig 4C). Altogether these data highlight the enrichment of fundamental nuclear protein functions in our nuclear extracts.

Different sets of proteins were analysed thoroughly in particular proteins governing the translational machinery and compared to the data obtained from PlasmoDB and Plasmobase.

Both 40S and 60S structural ribosomal proteins are known to be present in the nucleus [14] where the biogenesis of the 80S ribosomes takes place. We found 73 ribosomal proteins (29 and 44 proteins composing the 40S and 60S ribosomal sub-units, respectively). This number is in good agreement with the proteins found in higher eukaryotes [26]. The number of ribosomal proteins defined *in silico* in Plasmobase (78) and PlasmoDB (81) is greater than the number of proteins identified biologically (S6 Fig). PlasmoDB is currently curating the annotation of *Plasmodium* proteins, however since the scores of annotation are quite high in Plasmobase it confirms the annotation of most structural ribosomal proteins found in PlasmoDB.

Regarding proteins involved in translation (initiation, elongation and termination), we found protein expression evidence (S7 Fig) for 31 including one release factor among the 5 expected *in silico*. Both PlasmoDB (61) and Plasmobase (52) identified *in silico* more putative elongation factors and release factors that were not detected by the proteomics procedure. Worth mentioning the curation of *Plasmodium* proteins is a continuous ongoing process in PlasmoDB. In addition, several tRNA ligases and 3 signal recognition particles were found leading to 44 proteins.

We also focused on proteins involved in gene regulation of DNA and RNA synthesis. Several DNA replication proteins as well as DNA and RNA binding proteins were identified. Among the proteins involved in transcription only the very abundant proteins were easily detected, i.e. proteins involved in DNA remodelling including 7 histones (H2A, H2B, H3, H4 and variants), 2 nucleosome assembly proteins, 2 high mobility group B (HMGB1 and HMGB2) and heterochromatin protein 1 (HP1). However, only few specific transcription proteins (STP) were detected, that is in good agreement with our article dealing with the *in silico* survey of transcription associated proteins (TAP) [23] known to be at limiting concentration in the nucleus.

Finally, proteins with unknown function (UProt) were evaluated for new annotation (S8 Fig) with the help of the just released Plasmobase website [16]. Plasmobase provides significant information concerning domain architecture and co-occurrence helping annotation and proposition for protein function within 11 *Plasmodium* species. Some proteins can be found in all *Plasmodium* or be present in one species such as PF3D7_1468100 detected in *P*. *falciparum* only. The domain architectures can also be compared to proteins throughout evolution listed in UniProt Database. It is possible within the list of proteins from biologically well documented organisms (such as mammals, *Danio rerio*, several plants, fungi, mold or yeast) and sharing the same domain architectures to consider a function and propose an annotation for the corresponding *P*. *falciparum* protein (S8 Fig). This was done for UProt for which some putative protein annotations were proposed. Table 1 propose an example of annotation of nine UProt. Within the proteins listed, two proteins identified with a high score in Plasmobase were recently updated confirming PlasmoDB annotation and highlighting the efficacy of Plasmobase, whereas seven were not annotated in PlasmoDB. Plasmobase identified functional domains allowing putative annotations. Indeed, these functional domain architectures were explored in the UniProt Database a curated collection of manually evaluated and functionally annotated proteins eliciting experimental evidence at the level of proteins in well documented eukaryote species and associated to publications. Here, we show several putative proteins involved in RNA and translation regulation as well as transcriptional regulation. Indeed, functional verification is needed to validate the *in silico* annotation as for all putative *Plasmodium* proteins.

**Table 1:** Proposed functional annotation of some unknown proteins found in *P*. *falciparum* nuclear extracts.

ID	PlasmoDB	PlasmoBase	Explore Architecture		Domain description	Proposed protein Annotation	Protein with Experimental evidence	Publications
	Annotation	Domain	Plasmobase	Uniprot			in eukaryotes	
**PF3D7_0108500**	NO	ELM2	All *Plasmodium*	Metazoa Homo sapiens	N terminus of Myb1 and GATA transcription factors	Member of the ELM2 DNA binding protein family	http://www.uniprot.org/uniprot/Q6PJG2	https://www.ncbi.nlm.nih.gov/pubmed/25653165
**PF3D7_0519700**	Just updated	Fop_duplication	All *Plasmodium*	Metazoa Homo sapiens	Heterochromatin-associated protein & RNA processing	Chromatin associated protein family	http://www.uniprot.org/uniprot/Q9Y3Y2	https://www.ncbi.nlm.nih.gov/pubmed/20688955
**PF3D7_0618200**	NO	eIF3j Sub unit	All *Plasmodium*	Metazoa Homo sapiens	eIF3 subunit of the eIF-3 initiation complex	Member of the eIF3 translational protein complex	http://www.uniprot.org/uniprot/O75822	https://www.ncbi.nlm.nih.gov/pubmed/25849773
**PF3D7_0813300**	NO	nucleoplasmin	All *Plasmodium*	Metazoa Homo sapiens	chromatin de-condensation protein	Member of histone chaperone protein family	http://www.uniprot.org/uniprot/Q86SE8	https://www.ncbi.nlm.nih.gov/pubmed/21863821
**PF3D7_1147300**	NO	PrmA	All *Plasmodium*	Metazoa Homo sapiens	Ribosomal protein L11 methyltransferase	Member of the methyl transferase family	http://www.uniprot.org/uniprot/Q9NVM4	https://www.ncbi.nlm.nih.gov/pubmed/15044439
**PF3D7_1218500**	Just updated	dynamin_N	All *Plasmodium*	Metazoa Homo sapiens	Involved in the scission of a wide range of vesicles and organelles	Member of the dynamin superfamily	http://www.uniprot.org/uniprot/O60313	https://www.ncbi.nlm.nih.gov/pubmed/20038677
**PF3D7_1457300**	NO	MA3 x3	All *Plasmodium*	Metazoa Drosophila melanogaster	MI domain (after MA-3 and eIF4G), present in translation factors	Member of the eIF4G initiation translation family	http://www.uniprot.org/uniprot/Q53EL6	https://www.ncbi.nlm.nih.gov/pubmed/16357133
**PF3D7_1468100**	NO	Kelch 3, 4, 5, 6,	*Only P*. *Falciparum*	Metazoa Xenopus laevis	Kelch repeat domains involved in protein-protein intercation	Member of the Kelch repeat protein super family	https://www.uniprot.org/uniprot/G1X605 *	
**PF3D7_1249100**	NO	THUMP x2	All *Plasmodium*	Metazoa Homo sapiens	RNA binding domain protein involved in RNA modification	Putative member of RNA binding and modiying proteins	http://www.uniprot.org/uniprot/Q9NXG2	https://www.ncbi.nlm.nih.gov/pubmed/25653167

*Experimental evidence at transcript level

## Discussion

Our aim was to increase our knowledge about the protein content of the nucleus in *Plasmodium falciparum*. In this attempt, we conducted a large-scale proteomic analysis of 3D7 *P*. *falciparum* nuclear extracts obtained from unsynchronized cultures of parasitized erythrocytic cells. The use of mixed populations of the parasite gives access to all nuclear proteins whatever the stage.

The quality of our nuclear extract preparation was validated by the comparison of the protein contents of cytoplasmic and nuclear extracts (Figs 1B and S2). Indeed, proteins clearly known to be located in the nucleus such as epigenetic proteins (histones, HMGB and nucleosome remodeling proteins), RNA/DNA binding proteins and several TAP were present in the nuclear fraction only. In contrast, expected cytosolic proteins such as HSP60 recognized as a genuine cytosolic protein (S2 Fig) were absent from this fraction. The GO terms analysis further validates the good quality of our nuclear extract preparation and shows functional enrichment in translation, transcription and DNA replication processes. Of note, gene ontology annotations are far from being complete, a fortiori for *Plasmodium falciparum* organism. Thus, GO term data analysis should be taken with care. Nevertheless, several proteins were found within the two compartments. Even though this observation can be explained by some contamination of the nuclear extracts by cytoplasmic proteins this might be amplified by a continuous protein shuttle from cytoplasm to nucleus and *vice versa*. Many examples of dual localization of proteins in the nucleus and in the cytoplasm highlight the bi-directional shuttling of proteins including proteins involved in gene regulatory functions [5]. Interestingly, the structural ribosomal proteins are synthesized in the cytoplasm like all proteins but traffic back to the nucleus where ribosome biogenesis takes place [27].

To get deep into the nuclear proteome, we used two complementary proteomic approaches based on different fractionation strategies. In the first one, GeLC-MS/MS, proteins were fractionated according to their molecular weight by SDS-PAGE then fractions were submitted to tryptic digestion and peptides were analysed by reverse phase LC-MS/MS. In the second one, 2D-LC-MS/MS, proteins were first digested altogether then peptides were submitted to 2D liquid chromatography separation according to their native charge by strong cation exchange and to their hydrophobicity by reverse phase chromatography (Figs 3 and S4). Moreover, to reduce the competition effect between high and low abundance peptides, we used a precursor ion exclusion strategy (F.PIE and E.PIE) in 2D-LC-MS/MS experiments. This precursor exclusion procedure [20] [21] was used to reject the abundant peptides and therefore increase the detection number of low abundance proteins. Fig 2 shows an increased number of identified proteins of 30% (S3 Fig) with several additional proteins dedicated to overall gene regulation. Finally, these two methods proved to be complementary [4] [28]. Clearly, combining a MudPIT analysis of different biological NE replicates with an SDS-PAGE gel slice experiment gives the greatest amount of protein identification information from a limited amount of samples since many identified proteins were found with one method only. This is due to differential distribution of peptides in all different fractions and therefore modified peptide competition effects during reverse phase LC-MS/MS runs. Therefore, 446 proteins were obtained by combining the two approaches (see the complete list of proteins in S4B Fig).

Only one report appeared some years ago dealing with *P*. *falciparum* nuclear proteins [13]. Oehring *et al*. described the nuclear proteome at three different blood stages, namely schizonts, trophozoites and ring stages, identifying a total of 798 proteins. This highest number of proteins can be explained by the use of a mass spectrometer with higher resolution and mass accuracy (LTQ Orbitrap versus HCT ultra ion trap). Nevertheless, both studies are complementary since our study brings new nuclear proteins which had never been characterized before.

For instance, we found 73 structural ribosomal proteins involved in the 40S and 60S sub-units (S6 Fig) when Oehring *et al*. identified 67 proteins. The number of 73 is in good agreement with the 73 proteins found in human 40S and 60S sub-units [26]. *In silico* identified ribosomal proteins were 78 in Plasmobase and 81 in PlasmoDB. The high number of ribosomal proteins predicted in *Plasmodium* as regards to that reported for mammals might be due to a peculiarity of the parasite since diverse functional ribosomes were proposed in different development stages of the two hosts (mosquito and mammal) [29] or to bad annotation under active curation in GeneDB. For now, the identification of these 73 ribosomal proteins in *P*. *falciparum* nucleus validates both their protein existence and the intra-nucleus biogenesis of the two ribosomal sub-units in *Plasmodium*. These two ribosomal sub-units are further exported to the cytoplasm to govern protein synthesis [3].

Even though translational regulation remains a matter of interest for the *Plasmodium* community [30] [31], the proteins per se were poorly investigated [32]. Thirty-one proteins involved in translation (initiation, elongation and termination) were identified by Mass Spectrometry including one release factor among the 5 expected *in silico* when 28 proteins were identified by Oehring *et al*. [13] with no release factor (S7 Fig). Actually, PlasmoDB (61) and Plasmobase (52) listed more elongation and release factors proteins that were not detected by any of the two mass spectrometry procedures. Since, messenger RNA as well as ribosomal proteins and proteins involved in protein translation including initiation, elongation and termination factors have been detected in the nucleus, it has been hypothesized that in eukaryotes translation might occur in the nucleus. Actually, there are reports either in favour or against nuclear translation and this scenario remains a conflictual matter [15]. Our results add evidence to the nuclear localization of proteins involved in translation in eukaryotic cells.

Identification of *Plasmodium* proteins remains unsatisfactory since around half of the 5500 predicted Open Reading Frames awaits at least *in silico* functional annotation. As we are interested in proteins involved in *Plasmodium* gene regulation, we decided to identify the proteins found in 3D7 *P*. *falciparum* nucleus with a focus on proteins governing overall gene regulation. The number of general and epigenetic transcription factors predicted *in silico* still pending functional validation appears close to those described in higher eukaryotes [23]. However, we could not detect many transcription proteins like ApiAp2 proteins reported by Oehring *et al*. Additionally, the apparent small number of specific transcription protein (STP) apart from the ApiAp2 family stated in *Plasmodium* might be due to unsuccessful annotation owing to either AT rich genome and unusual codon usage or to yet undescribed transcription factors governing the parasite gene expression. Indeed, like in other eukaryotes, the proteins involved in the on and off switch that modulate gene regulation are most probably of low abundance. We are aware that the pretty low abundant proteins expressed in a small time range throughout the erythrocytic *P*. *falciparum* development such as the transcription factor Myb1 [33], might be highly diluted and therefore difficult to identify from these NE preparations. Actually, we were not able to detect the first described *Pf*Myb1 STP [33] nor did Voss’ group although we could assign its biological function in transcription [34]. This absence of PfMyb1 detection highlights the difficulty to identify other proteins than the well documented family of ApiAp2 in nuclear extracts [13]. However, we could detect another member of the Myb family (*Pf*Myb2) also identified by Voss’ group.

Finally, a number of unknown proteins are found in the nucleus synthesized from the corresponding open reading frames and addressed to the nucleus suggesting that some might participate to gene regulation. Using the Plasmobase website based on domain architectures and domain co-occurrence found in *Plasmodium* we could find in other eukaryotic species proteins with similar architectures listed in UniProt database which biological functions were investigated to propose an annotation (Table 1 and S8 Fig). We considered, within the unknown proteins found in NE the one sharing similar domain architectures described in UniProt eliciting mRNA and protein evidence with an assigned validated and published biological function. Thus, it was possible to propose a putative annotation for several *Plasmodium* including members of translation factor and DNA and RNA proteins associated to gene regulation.

The probable low abundance of specific transcription factors that have to be switched on and off rapidly to govern gene expression all along the *Plasmodium* complex live cycle from marked to weak parasite multiplication might be responsible for the challenging identification of these proteins. Actually, the number of identified transcription factors in nuclear extracts remained low compared to the list of PlasmoDB and Plasmobase and more work should be completed by using more sensitive MS devices. Finally, by taking advantage of Plasmobase website ability to annotate *Plasmodium* proteins it is reasonable to assume that in a next future we should be able to identify i) proteins already described in eukaryotes but difficult to annotate easily since their amino acid sequences are too divergent and ii) specific novel *Plasmodium* proteins involved in gene regulation.

## Supporting information

S1 Fig Schematic representation of the number of ions which can be excluded via the different precursor ion exclusion (PIE) procedures. (DOCX)

S2 Fig Pivot table presenting the comparative analysis of cytoplasmic and nuclear extracts. (XLS)

S3 Fig Pivot table presenting the comparative analysis of one nuclear extract NE2 analysed by 2D-LC-MS/MS (WE) associated to precursor ion exclusion (F.PIE and E.PIE). (XLSX)

S4 Fig A. Pivot table presenting the data obtained for all nuclear extracts analysed either by 2D-LC-MS/MS or GeLC-MS/MS. B. List of whole proteins obtained from all technical and biological replicates carried out with the two methods.
(XLSX)

S5 Fig Go terms of proteins present in nuclear and cytoplamic extracts. (XLSX)

S6 Fig Pivot table presenting the comparison of the ribosomal structural proteins obtained by the two biological procedures; our results (Briquet) and those of Till Voss’ team (Voss) and the data coming from PlasmoDB and Plasmobase website. (XLSX)

S7 Fig Pivot table presenting the comparison of the proteins of the mRNA translation machinery involved in initiation, elongation and termination obtained by the two biological procedures; our results (Briquet) and those of Till Voss’ team (Voss) and the data coming form PlasmoDB and Plasmobase website. (XLSX)

S8 Fig Annotation proposition for some unknown proteins. (XLSX)
