# Supplementary material for: Identification of Plasmodium falciparum nuclear proteins by mass spectrometry and proposed protein annotation
Source: PLoS One. 2018 Oct 31;13(10):e0205596. doi: 10.1371/journal.pone.0205596 (PMC6209197; doi:10.1371/journal.pone.0205596)
Supplement: S1 Fig — (DOCX) [file pone.0205596.s001.docx]

**S1 Appendix**

Number of peptides

**Nuclear extracts**

LC MS MS without exclusion **(WE)**

PIE precursor ion exclusion list generated from

the first data **(WE)**

Exclusion by fractions

Exclusion by fractions & retention times

39 x11 fractions

429 peptides

429 x5 retention times up to 2145 peptides

**[E. PIE**]

**[F. PIE]**
